# Optimising nutrition to improve growth and reduce neurodisabilities in neonates at risk of neurological impairment, and children with suspected or confirmed cerebral palsy

**DOI:** 10.1186/s12887-015-0339-2

**Published:** 2015-03-17

**Authors:** Morag J Andrew, Jeremy R Parr, Chris Montague-Johnson, Oliver Braddick, Karen Laler, Nicola Williams, Bonny Baker, Peter B Sullivan

**Affiliations:** 1 Department of Paediatrics, Oxford University, Level 2, Oxford Children’s Hospital, Oxford, OX3 9DU UK; 2 Institute of Neuroscience, Newcastle University, Sir James Spence Institute, Royal Victoria Infirmary, Newcastle Upon Tyne, NE1 4LP UK; 3 Department Experimental Psychology, University of Oxford, South Parks Road, Oxford, OX1 3UD UK; 4 Centre for Statistics in Medicine, University of Oxford, Botnar Research centre, Windmill Road, Oxford, OX3 7LD UK

**Keywords:** Docosahexaenoic acid, Choline, Uridine-5-monophosphate, Neonates, Infant, Neurodisability, Neurodevelopment, Cerebral palsy, Brain plasticity, Growth

## Abstract

**Background:**

Neurological impairment is a common sequelae of perinatal brain injury. Plasticity of the developing brain is due to a rich substrate of developing neurones, synaptic elements and extracellular matrix. Interventions supporting this inherent capacity for plasticity may improve the developmental outcome of infants following brain injury. Nutritional supplementation with combination docosahexaenoic acid, uridine and choline has been shown to increase synaptic elements, dendritic density and neurotransmitter release in rodents, improving performance on cognitive tests. It remains elusive whether such specific ‘neurotrophic’ supplementation enhances brain plasticity and repair after perinatal brain injury.

**Methods/Design:**

This is a two year double-blind, randomised placebo controlled study with two cohorts to investigate whether nutritional intervention with a neurotrophic dietary supplement improves growth and neurodevelopmental outcomes in neonates at significant risk of neurological impairment (the D1 cohort), and infants with suspected or confirmed cerebral palsy (the D2 cohort).

120 children will be randomised to receive dietetic and nutritional intervention, and either active supplement or placebo. Eligible D1 neonates are those born <30^+6^ weeks gestation with weight <9^th^ centile, ≤30^+6^ weeks gestation and Grade II, III or IV Intra-Ventricular Haemorrhage or periventricular white matter injury, or those born at 31-40^+28^ weeks gestation, with Sarnat grade I or II or III Hypoxic Ischaemic Encephalopathy or neuroimaging changes compatible with perinatal brain injury. Eligible D2 infants are those aged 1-18 months with a suspected or confirmed clinical diagnosis of cerebral palsy. The primary outcome measure is composite cognitive score on the Bayley Scales of Infant and Toddler Development III at 24 months. Secondary outcomes include visuobehavioural and visual neurophysiological assessments, and growth parameters including weight, height, and head circumference.

**Discussion:**

This is the first study to supplement neonates and infants with perinatal brain injury with the combination of factors required for healthy brain development, throughout the period of maximal brain growth. A further study strength is the comprehensive range of outcome measures employed. If beneficial, supplementation with brain phosphatide precursors could improve the quality of life of thousands of children with perinatal brain injury.

**Trial registration:**

Current Controlled trials: ISRCTN39264076 (registration assigned 09/11/2012), ISRCTN15239951 (registration assigned 23/04/2010).

## Background

Perinatal brain injury (PBI) has a range of consequences dependent upon the exact timing, location and extent of the brain insult. Brain white matter appears particularly vulnerable to injury in the preterm period [1], whereas grey matter injury is typically the consequence of acute injury sustained around term [2]. It is now recognised, however, that white and grey matter injury occur concurrently in pre-term and term brain injury [3,4]. Cerebral palsy (CP) is a common sequelae of brain injury in the antenatal, perinatal or postnatal period. CP describes “a group of permanent disorders of movement and posture, causing activity limitation that is attributed to non-progressive disturbances that occurred in the developing fetal or infant brain. The motor disorders of CP are often accompanied by disturbances of sensation, perception, cognition, communication, and behaviour, by epilepsy, and by secondary musculoskeletal problems” [5]. Although the brain injury resulting in CP is non-progressive, its manifestations vary throughout development. The incidence of CP worldwide is between 2.0-2.5 per 1000 live births [6,7], and CP is more common in infants born preterm, and in growth restricted infants [7]. Perinatal brain injury does not inevitably result in CP; however a range of cognitive and visual impairments are common sequelae, even in those who do not have severe motor impairment [8-10].

### Brain plasticity and functional recovery

Brain plasticity describes a process whereby the brain is functionally altered [11]. Studies of brain growth and development suggest that, as a result of an abundance of developing neurons and synaptic connections, the brain’s capacity for plasticity is probably highest through mid-gestation to early childhood [12]. It may also be true that the substrate richness that underlies the developing brain’s potential for plasticity also underlies its’ early vulnerability. Moreover, the outcome of perinatal brain injury may depend on the precise timing of injury; insults occurring during a “critical period” of development are more likely to cause significant impairment [12,13]. By contrast, insults occurring toward the end of specific developmental windows when critical neural connections have been made are less likely to result in as severe impairment [14].

The advent of more advanced neurophysiological and neuroimaging techniques has provided direct evidence of neuronal plasticity in infants and children [15]. For example, in children with congenital hemiparesis developing axons have been shown to re-route around areas of damage to reach their intended cortical target [16]. Furthermore, in children with congenital hemiparesis intensive therapy programmes can increase the size of primary hand motor cortex motor contralateral to the paretic hand, with associated functional improvement in the paretic hand [17-20]. Similar evidence for plasticity has also been demonstrated in the visual system where diffusion tractography imaging clearly identifies thalamo-cortical axon circumnavigation of white matter damage, to reach their predetermined destination in the occipital cortex [21].

Evidence supporting the plasticity potential of the developing brain raises the question of whether interventions that support the regeneration of developing neuronal projections might improve function as well as structure, and reduce neurodisability.

### Nutrition and brain development

The integrity and function of the brain depends in large measure upon a specific profile of membrane lipids and their fatty acids. In the brain, many enzymes involved in neurotransmitter metabolism are lipid-dependant, as are functions involved in the synthesis of brain structural lipids. Phosphatidylcholine is the primary component of neuronal cell membranes. The synthesis of brain phosphatidylcholine utilises three circulating precursors: choline; a pyrimidine e.g. uridine-5^1^-monophosphate (UMP); and a polyunsaturated fatty acid (PUFA) e.g. docosahexaenoic acid (DHA). Phosphatidylethanolamine, another component of neuronal cell membranes, may utilise two of these precursors, a pyrimidine and a PUFA. There is evidence in animals and humans that dietary supplementation of the precursors of neuronal cell membranes improves function. Dietary supplementation of rodents with a combination of DHA, choline and UMP increases brain phosphatide levels [22], pre and post-synaptic elements [22,23], and dendrite spine density [24], and is associated with improved performance on cognitive tasks [25]. Dietary supplementation of rats with UMP increase striatal levels of the neurotransmitter acetylcholine, relevant to cognitive processes in humans and other animals [26]. The first human randomised placebo controlled trial of DHA, choline and UMP has shown improvements in verbal recall on the Weschler Memory Scale-revised in adults with early Alzheimer’s disease [27].

### Nutrition and infant development

Infant milk formulae and normal feeding recommendations have been developed considering growth in weight and length as the main outcomes. Recently there been more consideration of neural development as an additional outcome when determining the optimal nutrition for neonates and infants.

Studies of improved early nutrition in preterm infants have shown improved neurodevelopmental outcomes in intervention groups [28,29]. Whether increased nutritional intake in the first years of life in children with CP improves growth or neurodevelopmental outcome, however, has not been adequately studied. Recently, a small non randomized trial of children with neurological impairments aged 1 – 14 years showed that those who received 6 months of additional nasogastric tube feeds, had improved growth and gross motor functioning compared with a group who did not receive supplements [30]. A further study identified increased head circumference and corticospinal tract diameter in infants fed with 120% of the recommended daily allowance, however neurodevelopmental outcome data was not reported [31].

The crucial role of long chain polyunsaturated fatty acids (LCPUFA) in infant brain development has been extensively investigated over the last decade. Studies have provided inconclusive results of the potential neurodevelopmental benefits of LCPUFA supplementation, in preterm and term infants. This may be a result of variation between conducted studies in design, maturity of supplemented infants, supplementation dose and duration, and outcome measures used. A recent individual patient data meta-analysis failed to show neurodevelopmental advantage of LCPUFA supplementation [32], but did not include data from two relevant studies which demonstrated improved performance on the Bayley Scales of Infant Development (BSID) in pre-term [33] and term infants [34]. The DINO study was the first trial to supplement preterm infants with levels of DHA approaching maximal concentrations found in human breast milk (around 1% total fatty acids) [35]. Infants were supplemented until discharge from hospital. The results of this randomised control trial (RCT) did not show any difference in the incidence of CP, but did demonstrate improved cognitive performance in preterm girls (but not boys), and in infants with birth weight less than 1250g in unadjusted analyses. Visual acuity was also improved in cases at 4 months compared to controls [35].

Whether or not the provision of adequate supplies of the biochemical precursors of neuronal cell membranes such as choline, uridine and DHA can improve neurological function in brain damaged infants remains unknown and has not been studied in infancy and childhood.

## Methods/Design

The overall aim of this study is to investigate whether nutritional intervention providing adequate choline, uridine and DHA supports the plasticity potential and regeneration of neuronal projections in the developing brain, to improve function and structure, and reduce neurodisability.

### Primary objective

To investigate whether nutritional intervention, with supplementation of choline, uridine and DHA improves neurodevelopmental outcomes in neonates at significant risk of neurological impairment, and infants with suspected or confirmed CP.

### Secondary objectives

To investigate whether nutritional intervention improves growth, visual outcomes and improved indices of general health status, including prevalence of epilepsy, feeding difficulties, clinically significant gastro-oesophageal reflux, constipation, chest infections (requiring antibiotics) and hospital admissions.

### Primary outcome measure

The primary outcome measure is performance on the composite cognitive scale of the BSD- III following 24 months of supplementation.

### Secondary outcome measures

- BSID-III composite cognitive scale at 12 months
- BSID-III composite language score at 12 and 24 months
- BSID-III composite motor score at 12 and 24 months
- Visual function, including Pattern Reversal Visual Event Related Potential (PR-VERP) latency and behavioural vision assessment score at 12 and 24 months
- Growth
  - ◦ Weight for age z-score at 12 and 24 months
  - ◦ Length for age z-score at 12 and 24 months
  - ◦ Triceps skinfold thickness for age z-score at 12 and 24 months
  - ◦ Mid-upper arm circumference for age z-score at 12 and 24 months
  - ◦ Head circumference for age z-score at 12 and 24 months

### Study design

The Dolphin studies are double-blind randomised placebo-controlled trials of supplementation with a combination of DHA, choline and uridine. Two cohorts will be recruited:

- *Dolphin 1 (D1): Optimising nutrition to improve growth and reduce neurodisabilities in neonates at risk of neurological impairment* and
- *Dolphin 2 (D2): Optimising nutrition to improve growth and reduce neurodisabilities in children with suspected or confirmed cerebral palsy.*

### Subject population

Term and pre-term neonates who are at significant risk of neurological impairment, either as the result of a perinatal neurological event or by their extreme prematurity and low birth weight (<9^th^ centile).

Infants up to 18 months of age with a suspected or confirmed diagnosis of CP, made by their consultant paediatrician.

### Neonatal inclusion criteria

Birth ≤30^+6^ weeks gestation and:

- Small for Gestational Age – weight less than 9th centile OR
- Sarnat Grade IIa, IIb, III or IV Germinal Matrix Haemorrhage (GMH) – Intra Ventricular Haemorrhage (IVH) or pathological periventricular flare/ leucomalacia

Birth 31-40^+28^ weeks gestation:

- Hypoxic Ischaemic Encephalopathy Sarnat Grade II and III OR
- Grade IIa, IIb, III or IV GMH-IVH or pathological periventricular flare/ leucomalacia OR
- Magnetic resonance imaging (MRI) abnormalities: Posterior limb of the internal capsule (PLIC), basal ganglia, thalami, white matter and cortex.

Consent to enter the trial will be obtained before the infant is 4 weeks post term corrected age.

### Infant inclusion criteria

Aged 1–18 months with a suspected or confirmed clinical diagnosis of cerebral palsy according to their consultant Paediatrician, using the following definition:

*“A group of permanent disorders of the development of movement and posture, causing activity limitations that are attributed to non-progressive disturbances that occurred in the developing fetal or infant brain. The motor disorders of cerebral palsy are often accompanied by disturbances of sensation, perception, cognition, communication, and behaviour, by epilepsy, and by secondary musculoskeletal problems”* [5].

### Exclusion criteria

- ◦ Progressive neurological conditions
- ◦ Gastrointestinal disease which significantly impairs absorption
- ◦ Multiple congenital abnormalities or syndromic associations
- ◦ Such low hearing that assessment with the Bayley scales cannot be completed
- ◦ Parents considered by clinicians to be unable to follow study protocol

And for infants born at <36 weeks gestation:

- ◦ Grade I GMH-IVH

### Identification and recruitment of participants

This is a multi-centre trial to be carried out at the Oxford University Department of Paediatrics with 6 collaborating regional hospitals. Participants will be recruited following referral from Neonatologists, Community Paediatricians and Neurologists at these collaborating sites.

#### Neonatal identification

Term and pre-term neonates will be recruited from the neonatal units at the Oxford University Hospitals NHS Trust, Oxford, the Royal Berkshire Hospital, Reading, and Wexham Park Hospital, Slough. Eligible neonates will be identified by the neonatal clinical team who make the initial parental approach, with support from the research team. The trial will only be discussed with the parents when their baby is fully enterally fed and no longer receiving neonatal intensive care. With parental agreement the research nurse (CMJ) or clinical research fellow (MA) will then approach the family to discuss the trial in more detail and give them the parent written information.

#### Infant identification

Paediatricians and Neurologists with knowledge of the infant’s previous clinical history, presenting clinical condition, and study protocol will identify infants with a clinical diagnosis of suspected or confirmed cerebral palsy at the collaborating research sites. These sites are at Stoke Mandeville Hospital and Wycombe General Hospitals (Buckinghamshire Hospitals NHS Trust), Northampton General Hospital (Northampton General Hospital NHS Trust), Department of Child Health (Milton Keynes Hospital NHS Foundation Trust), The Child Development Centres at Upton Hospital, and St Marks Hospital, (Heatherwood and Wexham Park Hospitals NHS Foundation Trust), and the Dingley Specialist Children’s Centre at the Royal Berkshire NHS Foundation Trust. With the lead clinicians’ agreement, the clinical research fellow (MA) or research nurse (CMJ) will contact the parents by telephone to explain the trial in more detail and if they are interested, send them written information.

### Consent procedures

#### Neonates

The research nurse or clinical research fellow will take written consent, either on the neonatal unit or, if the baby had been discharged subsequent to the written information being given, at the family home.

#### Infants

Written consent will be taken by the research nurse or clinical research fellow during a pre-arranged visit to the family home.

### Randomisation and blinding

Randomisation will be carried out by the study statistician (NW) in the University of Oxford Centre for Statistics in Medicine, Oxford (CSM). Randomisation will occur following consent, at least 24 hours before start of supplementation. Both neonates and infants will be randomised to intervention group X or Y, to receive dietetic and nutritional intervention, with either active or placebo supplementation. The randomisation programme will include a minimisation algorithm which will be used to ensure balanced distribution of children with different severities of brain injury between intervention groups. The research team will be notified by CSM of randomisation group by return fax of the completed randomisation form.

#### Neonate stratification

Neonates will be allocated across the 2 treatment groups by the following prognostic factors: gender, gestational age (≤30^+6^ weeks, ≥31 weeks) and severity of brain injury (normal/mild, moderate, severe – see Table 1).

**Table 1:** **Neuroimaging severity categorisation** [2,49-57]

	Normal/Mild	Moderate	Severe
Preterm injury			
• Cranial ultrasound scan (cUSS)	• Normal	• Grade III IVH	• Grade IV IVH
	• Grade I/II Intraventricular haemorrhage (IVH)	• Non-cystic Periventricular leucomalacia (PVL)	• Periventricular haemorrhage infarction (PVHI)
	• Ventricular index (VI) < 13 mm Term equivalent age (TEA) OR	• VI 13-15 mm TEA OR	• Cystic PVL
	• VI < 97^th^ percentile for corrected gestational age (CGA)	• VI >97^th^ percentile but < 4 mm above 97^th^ percentile for CGA	• Subcortical leucomalacia
			• VI at TEA >15 mm OR
			• VI >4 mm above 97^th^ percentile for CGA
			• Basal ganglia (BG) lesions
			• Focal infarction
Term hypoxic ischaemic encephalopathy			
• Magnetic resonance imaging (MRI)	• Focal subtle abnormalities of BG with normal appearance of the posterior limb of the internal capsule (PLIC)	• Multi-focal lesions in BG with equivocal or abnormal signal intensity within PLIC	• Widespread abnormalities involving all Basal ganglia-Thalamus (BGT) structures and PLIC
• cUSS where MRI unavailable	• Periventricular white matter changes difficult to differentiate from normal appearances and therefore not classified as abnormal	• Small focal lesions of without loss of grey matter (GM)/WM differentiation.	• Larger areas of abnormality with loss of GM/WM differentiation, consistent with infarction
	• Changes confined to cerebral cortex and subcortical white matter (WM)		• Central grey matter hyperechogenicity +/− more extensive cortical and subcortical hyperechogenicity
Term infarction			
• MRI (cUSS where MRI unavailable)		• Focal, non-territorial infarct	• Territorial infarct

#### Infant stratification

Infants will be stratified by gender, age at recruitment (1–5 months, 6–12 months, and 13–18 months), known visual impairment at trial entry and severity of motor disorder (4 limb involvement or other).

The research team will be blinded to treatment group allocation with the exception of the trial dietician (KL) and trial assistant (BB). KL will responsible for the delivery of supplement to participating families and will be aware of group allocation (X or Y), as will be participating families. Unblinding at the end of the trial will be undertaken by CSM.

### Intervention

Parents will receive fortnightly input from the trial dietitian (KL) to optimise macro- and micro-nutrient intake. Each infant’s diet will be supplemented with 2g/kg/day of study product for 2 years. The supplement will be added to the infant’s milk or mixed with solids depending on the age and diet of individual participants. Detailed advice on dose incrementation and supplement mixing with food or fluid will be provided by the trial dietician (KL). The active supplement contains DHA (1% total fatty acids), arachidonic acid (AA), choline, UMP, multivitamins, trace elements and minerals. The placebo contains multivitamins, minerals and trace elements only. Supplement sachets are labelled X or Y and are available in 2g, 3g and 12g quantities. The supplements are produced under strict Good Manufacturing Practice (GMP) conditions and donated by Nutricia®, Netherlands, and have been deodorised to minimise smell and taste. Parents and assessors will be blinded as to whether X or Y is the active supplement.

### Study assessments

A detailed schedule of the study assessments is shown in Table 2.

**Table 2:** **Schedule of trial assessments**

Procedure	Frequency	Details
Feed supplementation	Daily	Both groups will receive a measured feed supplement (active or placebo) to add to a milk feed daily
Dietetic review	Every 2 weeks or as required	Dietetic review will take place in person or by telephone every two weeks or as required for the duration of the trial
Anthropometry	Every 3 months	Measurements will be taken using calipers and anthropometer at baseline and then every 3 months to monitor growth.
MRI/MRS	0 and 3 months	This scan will be performed at baseline and then three months later to assess brain chemistry and choline uptake.
Visual Event Related Potential and behavioural vision testing	Baseline, term, 6 m, 12 m, 24 m	During this test the child will be positioned on the parents lap or in a chair to view a monitor where moving black and white stripes were shown. For the test 3 adhesive electrodes will be placed on the head and connected to a computer by fine cables. The child will also be observed for reactions to moving stimuli and given simple tasks to perform
Bayley Assessment	Baseline, 12 and 24 months	The child will be asked to do a number of activities to see if their thinking, language, and moving (sitting, walking) skills are similar to children his or her own age.
Vineland Assessment	12 and 24 months	During a semi-structured interview the parents will be given a questionnaire to fill in about their child’s personal and social skills.
Fatty acid profile analysis	Baseline and 24 months	0.05mls of blood will be taken using a finger prick test.
Maternal fatty acid profile analysis	Baseline	0.05mls of blood will be taken using a finger prick test.

#### Treatment monitoring and growth outcomes

Head circumference, weight, length, height, mid-arm circumference and skinfold thickness (triceps, biceps, supra-iliac and sub-scapular) will be measured 3 monthly [36].

Participant whole blood fatty acid levels will be measured by heel or finger-prick [37] at trial entry and at the end of supplementation. In the neonatal trial maternal whole blood fatty acid levels will be measured. Blood fatty acid levels at baseline and trial end will be correlated with neurodevelopmental outcome.

#### Developmental assessment

The BSID II [38] has been widely used to determine rates of developmental disability or disordered development [39,40], and as an outcome measure in RCTs [41,42]. The BSIDIII [43] has superseded the BSID II and includes separate composite scores for cognitive, language and motor scales. The BSID III will be administered in the child’s home by a trained administrator (MA or CMJ). Assessments will be video-recorded for verification of scoring.

The Vineland Adaptive Behaviour Scales II (VABS-II) [44] is a standardised parental interview, and will be administered by MA or CMJ following Bayley assessment, as a parent report measure of child development.

#### Functional vision assessment

Vision assessment will be performed by MA and CMJ under the supervision of Professors O Braddick and J Atkinson. Assessments will be conducted in vision testing facilities in the Women’s Centre, Oxford University Hospitals NHS Trust, Oxford. Infants will sit on the caregiver’s lap during testing. The Atkinson Battery of Child Development for Examining Functional Vision (ABCDEFV) [45] will be used to assess perceptual, motor, spatial and cognitive skills, and published normative data are available [45].

Core vision tests will include orthoptic assessment (ocular movements, pupil response), refractive errors, binocular optokinetic nystagmus (OKN), acuity (Teller acuity cards), and attention at distance, visual fields (Stycar balls), defensive blink and fixation shift. Age-specific tests to identify problems in perceptual, visuo-motor and spatio-cognitive domains will be included.

#### Neurophysiological measures

##### Phase and orientation reversal visual event related potentials (PR- and OR-VERP)

During VERP recording infants sit in a darkened room on their caregiver’s lap, 40 cm from the stimulus screen. Three electrodes are placed on the infants scalp, over the occiput, forehead, and vertex. Electrodes are connected to a low voltage pre-amplifier. Stimuli are oblique black and white stripes. For phase reversal VERPs (PR-VERP) the stripe orientation is unchanged but contrast reverses periodically. For orientation reversal VERPs (OR-VERP) stripe orientation changes between 45 and 135 degrees. Both PR- and OR-VERPs are measured at transient (2 reversals/second) and steady state (4–8 reversals/second). 100 sweeps are recorded at each reversal rate.

##### Neuro-imaging

In the neonatal (D1) trial, brain proton magnetic resonance spectroscopy (1H-MRS) will be used to assess brain choline uptake in cases versus controls, at baseline and following 3 months of supplementation. MR studies cannot be performed beyond this age without sedation.

1H-MRS and MRI studies will be performed using a Philips 1.5 T Achieva machine, with an 8-channel SENSE adult head coil. 1H-MRS will be performed in natural sleep following a milk feed. In neonates and infants 1H-MRS will also be performed at the end of the intervention if MRI under general anaesthetic is performed for clinical reasons. Axial T1 and T2 weighted images will be obtained. T2 weighted images will be used to place two 1H-MRS slices, one at basal ganglia level, the other through the cerebral hemispheres above the lateral ventricles. 1H-MRS acquisitions will be performed using a manufacturer standard two-dimensional CSI technique. The volume of interest will be sized so that suppression bands (REST slabs) lie well within the inner table of the calvarium. After automated shimming and water suppression steps, spectra will be acquired for each of the 25 voxels of the 5 × 5 matrix within the sampled volume at each level. 1H-MRS data will be analysed using a Philips Extended Workspace with SpectroView software, which generates spectra for each voxel, performs metabolite peak fitting for choline, creatine, N-acetyl aspartate (NAA) and lactate, and calculation of corresponding metabolite ratios.

The infant (D2) trial participants will not undergo MRI/MRS as part of the protocol.

### Data collection, storage and record keeping

Each participant will be allocated a unique study number at the time of randomisation and this will be used throughout the study to identify all data relating to the participant. All assessment data collected by the research nurse, clinical research fellow or dietician will be entered on paper then transferred to a data protection compliant drive in the research team’s office. All data will be double entered and data entry errors corrected. Data stored on the database will be updated by the University’s Information Management Services Unit every night. All patient identifiable information will be kept on encrypted computers in a secure office, with access limited to authorised members of the research team. In accordance with the requirements of the University of Oxford, all data will be archived in secure archive facilities within the University until three years after the last participant reaches the age of eighteen. After this time all documentation will be destroyed and all data deleted securely. The final dataset will initially be available only to the study team. After the results of the trials have been reported, anonymous trial data will be made publicly available.

### Sample size calculation

No previous studies have supplemented children with this combination of micronutrients. Power calculations were performed for primary outcome measure BSID-III score, assuming power of 80%, 5% significance level, use of two-sided statistical tests throughout and equal allocation to each arm. Recruitment of 60 participants to each trial (30 to each arm within each trial), assuming 20% loss to follow-up, provided 80% power to detect a 12.5 point difference in BSID-III score assuming standard deviation (SD) of 15 points. Power calculations were also performed for secondary outcome VERP latency. 30 infants per group provided 90% power to detect a latency difference of 25 ms, assuming SD of 25 ms and significance level of 5%.

### Statistical analysis

Data analysis will be undertaken by the study statistician at CSM using appropriate statistical software.

Continuous variables at baseline will be presented using means and standard deviations (unless not normally distributed, in which case medians and percentiles will be used).

Categorical/binary variables at baseline will be presented using proportions and percentages.

The primary outcome (change in Bayley score from baseline) will be analysed at 12 and 24 months using mixed effects linear regression to account for the repeated measures over time. Baseline Bayley score will be entered as a covariate in the model. The mixed effects model will include Bayley score at 12 and 24 months as the response variable, time point (12 or 24 months), treatment group and baseline Bayley score as fixed effects and a patient specific random intercept. An interaction between time and treatment group will be fitted as a fixed effect to allow estimation of treatment effect at both time points. The minimisation (design) factors will also be included as fixed effects. The primary outcome is Bayley score at 24 months. Mean difference in cognitive score between the 2 groups at 24 months will be presented along with 95% CI and associated 2 sided p-value.

If the Bayley score has severe departure from normality, thus invalidating the linear regression model, the first approach will be transformation. If the data cannot be transformed to normality, a Mann–Whitney test will be adopted. The difference in median change from baseline will be presented alongside the 95% CI for the difference in medians. There will be no adjustment for covariates if the data cannot be transformed to allow parametric analyses. Primary analysis will be conducted on the Intention To Treat population.

All analyses of continuous outcomes will follow the same procedure as the primary outcome analysis. Analysis of proportions will use binomial regression. Odds ratios will be presented.

If missing data are substantial, multiple imputation will be used to assess the impact of missing data in a sensitivity analysis.

### Withdrawal of participants

Parents will have the right to withdraw their child from the study at any time, for any reason, and without giving a reason. The Chief Investigator will also have the right to withdraw participants from the study in the event of adverse events, serious adverse events, suspected unexpected serious adverse reactions or protocol violations. Where parents withdraw before randomisation, completion of baseline assessments and without starting supplementation, they will be replaced. If a parent decides to withdraw their child from the study every effort will be made to report the reason for withdrawal as thoroughly as possible. Parents will be informed, prior to consenting into the study, that should they subsequently withdraw all data collected prior to their withdrawal will be maintained and used for the purposes of the study.

### Management of the study, quality control and assurance

The study will be managed through the University of Oxford’s Clinical Trials Research Group and the research team of the Paediatric Gastroenterology and Nutrition Group in the Department of Paediatrics. Quality control will be maintained through adherence to the study protocols, standard operating procedures, research governance and clinical trial regulations.

An independent Data Monitoring and Ethics Committee, consisting of a senior medical statistician and two independent paediatric clinicians, will meet part-way through the study to provide independent review. It's purpose will be to safeguard the interests of trial participants, assess the safety and efficacy of the interventions, and monitor the overall conduct of the clinical study. The committee will be chaired by one of the independent clinicians and will have access to unblinded study data.

### Plans to communicate study results

No publications containing results from this study have been published or submitted to any journal. Results will be submitted to peer-reviewed journals for publication when all statistical analyses have been completed and the study unblinded. This should be in the summer of 2015.

The research team will contact all the participants when the results of the study are available. Results will be sent to all participants who expressed interest in receiving them.

### Ethics committee and regulatory approval

The study will be conducted in accordance with ethical principles as listed in the Declaration of Helsinki. Ethics approval was granted by Oxfordshire REC B for both trials. Approval for the neonatal trial was granted on 08 May 2008, number 08/H0605/70. Approval for the infant trial 12 January 2009, number 08/H0605/155. A total of 6 substantial amendments were submitted to the REC and approved as follows:

1st amendment - 19 February 2009

2nd amendment - 21 July 2009

3rd amendment - 7 January 2010

4th amendment - 13 May 2010

5th amendment - 10 August 2010

6^th^amendment - 13 November 2012.

## Discussion

This novel project will be the first to provide all three phosphatide precursors (DHA, choline and UMP) to infants at risk of neurodisability. There are very few interventions that have been shown to improve the neurodevelopmental outcome of babies and infants with PBI. To date therapeutic hypothermia for term hypoxic encephalopathy is the only therapy which improves mortality and morbidity outcomes [46]. There is a clear need to identify other treatments capable of reducing morbidity in infants who sustain perinatal brain injury. If successful, dietary supplementation with this combination of phosphatide precursors will ameliorate the level of disability experienced by these children and their families.

This study has several advantages over previous studies examining the effects of DHA supplementation on infant neurodevelopment. Firstly, it provides DHA at 1% total fatty acids, mimicking maximal levels found during third trimester transplacental fatty acid transfer [47] and human breast milk [48]. With the exception of the DINO trial [35], previous fatty acid supplementation studies have given DHA at much lower doses, and have not provided the additional substrates required for normal neural development. However, the combination of DHA with choline and UMP has been shown to have positive synergistic effects on brain phosphatide levels, synaptic elements and dendritic spine density compared to when these nutrients are given alone. This study will be the first to provide high dose DHA in combination with choline and UMP. Furthermore, in contrast with other studies, most of which had a short intervention period, this study will supplement infants for 2 years, throughout the period of maximal brain growth and synaptogenesis. This will be a longer period of supplementation than any previous DHA intervention trial, thus minimising the likelihood of type 2 errors resulting from an insufficient supplementation period. A further study strength will be the range of outcome measures being employed, utilising behavioural, neurophysiological and neuroimaging measures.

If this study demonstrates a neurodevelopmental advantage to supplementation with phosphatide precursors, the supplement could be provided as part of routine clinical care for babies with PBI, or the supplement’s effectiveness could be tested in a larger multi-centre trial.

### Study status

The study is in progress and will continue until the last infant recruited completes their 2 year participation. This is anticipated to be in March 2015. A period of statistical analysis will follow and it is anticipated that this will be completed in the summer of 2015.

## Abbreviations

PBI: Perinatal brain injury
CP: Cerebral palsy
PUFA: Polyunsaturated fatty acid
DHA: Docosahexaenoic acid
UMP: Uridine-5^1^-monphosphate
LCPUFA: Long chain polyunsaturated fatty acid
BSID-III: Bayley scales of infant development III
RCT: Randomised control trial
PR-VERP: Pattern reversal visual event related potential
GMH: Germinal matrix haemorrhage
IVH: Intra ventricular haemorrhage
MRI: Magnetic resonance imaging
PLIC: Posterior limb of the internal capsule
CSM: Centre for statistics in medicine
GMP: Good manufacturing practice
VABS-II: Vineland adaptive behaviour scales II
ABCDEFV: Atkinson battery of child development for examining functional vision
OKN: Optokinetic nystagmus
VERP: Visual event related potential
OR-VERP: Orientation reversal visual event related potential
MRI: Magnetic resonance imaging
1H-MRS: magnetic resonance spectroscopy
NAA: N-acetyl aspartate
SD: Standard deviation
cUSS: Cranial ultrasound scan
VI: Ventricular index
TEA: Term equivalent age
CGA: Corrected gestational age
PVL: Periventricular leucomalacia
PVHI: Periventricular haemorrhagic infarction
BG: Basal ganglia
WM: White matter
GM: Grey matter
BGT: Basal ganglia- Thalamus

## References

[1] Kostovic I, Jovanov-Milosevic N (2006). The development of cerebral connections during the first 20–45 weeks’ gestation. Semin Fetal Neonatal Med.

[2] Okereafor A, Allsop J, Counsell SJ, Fitzpatrick J, Azzopardi D, Rutherford MA (2008). Patterns of brain injury in neonates exposed to perinatal sentinel events. Pediatrics.

[3] Rees S, Harding R, Walker D (2011). The biological basis of injury and neuroprotection in the fetal and neonatal brain. Int J Dev Neurosci.

[4] Counsell SJ, Tranter SL, Rutherford MA (2010). Magnetic resonance imaging of brain injury in the high-risk term infant. Semin Perinatol.

[5] Rosenbaum P, Paneth N, Leviton A, Goldstein M, Bax M, Damiano D (2007). A report: the definition and classification of cerebral palsy April 2006. Dev Med Child Neurol Suppl.

[6] Surman G, Hemming K, Platt MJ, Parkes J, Green A, Hutton J (2009). Children with cerebral palsy: severity and trends over time. Paediatr Perinat Epidemiol.

[7] Oskoui M, Coutinho F, Dykeman J, Jette N, Pringsheim T (2013). An update on the prevalence of cerebral palsy: a systematic review and meta-analysis. Dev Med Child Neurol.

[8] Johnson S, Fawke J, Hennessy E, Rowell V, Thomas S, Wolke D (2009). Neurodevelopmental disability through 11 years of age in children born before 26 weeks of gestation. Pediatrics.

[9] Marlow N, Rose AS, Rands CE, Draper ES (2005). Neuropsychological and educational problems at school age associated with neonatal encephalopathy. Arch Dis Child Fetal Neonatal Ed.

[10] Perez A, Ritter S, Brotschi B, Werner H, Caflisch J, Martin E (2013). Long-term neurodevelopmental outcome with hypoxic-ischemic encephalopathy. J Pediatr.

[11] Pascual-Leone A, Amedi A, Fregni F, Merabet LB (2005). The plastic human brain cortex. Annu Rev Neurosci.

[12] Johnston MV (2009). Plasticity in the developing brain: implications for rehabilitation. Dev Disabil Res Rev.

[13] Schneider M, Koch M (2005). Behavioral and morphological alterations following neonatal excitotoxic lesions of the medial prefrontal cortex in rats. Exp Neurol.

[14] Kolb B, Gibb R (2011). Brain plasticity and behaviour in the developing brain. J Can Acad Child Adolesc Psychiatry.

[15] Inguaggiato E, Sgandurra G, Perazza S, Guzzetta A, Cioni G (2013). Brain reorganization following intervention in children with congenital hemiplegia: a systematic review. Neural plasticity.

[16] Staudt M, Gerloff C, Grodd W, Holthausen H, Niemann G, Krageloh-Mann I (2004). Reorganization in congenital hemiparesis acquired at different gestational ages. Ann Neurol.

[17] Sutcliffe TL, Logan WJ, Fehlings DL (2009). Pediatric constraint-induced movement therapy is associated with increased contralateral cortical activity on functional magnetic resonance imaging. J Child Neurol.

[18] Sterling C, Taub E, Davis D, Rickards T, Gauthier LV, Griffin A (2013). Structural neuroplastic change after constraint-induced movement therapy in children with cerebral palsy. Pediatrics.

[19] Cope SM, Liu XC, Verber MD, Cayo C, Rao S, Tassone JC (2010). Upper limb function and brain reorganization after constraint-induced movement therapy in children with hemiplegia. Develop Mental neurorehabilitation.

[20] Golomb MR, McDonald BC, Warden SJ, Yonkman J, Saykin AJ, Shirley B (2010). In-home virtual reality videogame telerehabilitation in adolescents with hemiplegic cerebral palsy. Arch Phys Med Rehabil.

[21] Guzzetta A, D’Acunto G, Rose S, Tinelli F, Boyd R, Cioni G (2010). Plasticity of the visual system after early brain damage. Dev Med Child Neurol.

[22] Wurtman RJ, Ulus IH, Cansev M, Watkins CJ, Wang L, Marzloff G (2006). Synaptic proteins and phospholipids are increased in gerbil brain by administering uridine plus docosahexaenoic acid orally. Brain Res.

[23] Cansev M, Marzloff G, Sakamoto T, Ulus IH, Wurtman RJ (2009). Giving uridine and/or docosahexaenoic acid orally to rat dams during gestation and nursing increases synaptic elements in brains of weanling pups. Dev Neurosci.

[24] Sakamoto T, Cansev M, Wurtman RJ (2007). Oral supplementation with docosahexaenoic acid and uridine-5′-monophosphate increases dendritic spine density in adult gerbil hippocampus. Brain Res.

[25] Holguin S, Martinez J, Chow C, Wurtman R (2008). Dietary uridine enhances the improvement in learning and memory produced by administering DHA to gerbils. FASEB J.

[26] Wang L, Albrecht MA, Wurtman RJ (2007). Dietary supplementation with uridine-5′-monophosphate (UMP), a membrane phosphatide precursor, increases acetylcholine level and release in striatum of aged rat. Brain Res.

[27] Scheltens P, Kamphuis PJ, Verhey FR, Olde Rikkert MG, Wurtman RJ, Wilkinson D (2010). Efficacy of a medical food in mild Alzheimer’s disease: a randomized, controlled trial. Alzheimers Dement.

[28] Lucas A, Morley R, Cole TJ (1998). Randomised trial of early diet in preterm babies and later intelligence quotient. BMJ (Clinical research ed).

[29] Lucas A, Morley R, Cole TJ, Gore SM, Lucas PJ, Crowle P (1990). Early diet in preterm babies and developmental status at 18 months. Lancet.

[30] Campanozzi A, Staiano A (2010). Impact of malnutrition on gastrointestinal disorders and gross motor abilities in children with cerebral palsy. Brain Dev.

[31] Dabydeen L, Thomas JE, Aston TJ, Hartley H, Sinha SK, Eyre JA (2008). High-energy and -protein diet increases brain and corticospinal tract growth in term and preterm infants after perinatal brain injury. Pediatrics.

[32] Beyerlein A, Hadders-Algra M, Kennedy K, Fewtrell M, Singhal A, Rosenfeld E (2010). Infant formula supplementation with long-chain polyunsaturated fatty acids has no effect on Bayley developmental scores at 18 months of age–IPD meta-analysis of 4 large clinical trials. J Pediatr Gastroenterol Nutr.

[33] Clandinin MT, Van Aerde JE, Merkel KL, Harris CL, Springer MA, Hansen JW (2005). Growth and development of preterm infants fed infant formulas containing docosahexaenoic acid and arachidonic acid. J Pediatr.

[34] Birch EE, Garfield S, Hoffman DR, Uauy R, Birch DG (2000). A randomized controlled trial of early dietary supply of long-chain polyunsaturated fatty acids and mental development in term infants. Dev Med Child Neurol.

[35] Makrides M, Gibson RA, McPhee AJ, Collins CT, Davis PG, Doyle LW (2009). Neurodevelopmental outcomes of preterm infants fed high-dose docosahexaenoic acid: a randomized controlled trial. JAMA.

[36] Stevenson RD (1995). Use of segmental measures to estimate stature in children with cerebral palsy. Arch Pediatr Adolesc Med.

[37] Marangoni F, Colombo C, Galli C (2004). A method for the direct evaluation of the fatty acid status in a drop of blood from a fingertip in humans: applicability to nutritional and epidemiological studies. Anal Bio chem.

[38] Bayley N (1993). The bayley scales of infant development.

[39] Hintz SR, Kendrick DE, Vohr BR, Poole WK, Higgins RD (2005). Changes in neurodevelopmental outcomes at 18 to 22 months’ corrected age among infants of less than 25 weeks’ gestational age born in 1993–1999. Pediatrics.

[40] Wood NS, Marlow N, Costeloe K, Gibson AT, Wilkinson AR (2000). Neurologic and developmental disability after extremely preterm birth. EPICure study group. N Engl J Med.

[41] Bouwstra H, Dijck-Brouwer DA, Boehm G, Boersma ER, Muskiet FA, Hadders-Algra M (2005). Long-chain polyunsaturated fatty acids and neurological developmental outcome at 18 months in healthy term infants. Acta Paediatr.

[42] Fewtrell MS, Morley R, Abbott RA, Singhal A, Isaacs EB, Stephenson T (2002). Double-blind, randomized trial of long-chain polyunsaturated fatty acid supplementation in formula fed to preterm infants. Pediatrics.

[43] Bayley N (2006). The bayley scales of infant and toddler development.

[44] Sparrow S, Cicchetti D, Balla D (2005). Vineland adaptive behavior scales.

[45] Atkinson J, Anker S, Rae S, Hughes C, Braddick O (2002). A test battery of child development for examining functional vision (ABCDEFV). Strabismus.

[46] Edwards AD, Brocklehurst P, Gunn AJ, Halliday H, Juszczak E, Levene M (2010). Neurological outcomes at 18 months of age after moderate hypothermia for perinatal hypoxic ischaemic encephalopathy: synthesis and meta-analysis of trial data. BMJ (Clinical research ed).

[47] Clandinin MT, Chappell JE, Heim T, Swyer PR, Chance GW (1981). Fatty acid utilization in perinatal de novo synthesis of tissues. Early Hum Dev.

[48] Brenna JT, Varamini B, Jensen RG, Diersen-Schade DA, Boettcher JA, Arterburn LM (2007). Docosahexaenoic and arachidonic acid concentrations in human breast milk worldwide. Am J Clin Nutr.

[49] Beaino G, Khoshnood B, Kaminski M, Pierrat V, Marret S, Matis J (2010). Predictors of cerebral palsy in very preterm infants: the EPIPAGE prospective population-based cohort study. Dev Med Child Neurol.

[50] de Vries LS, Van Haastert IL, Rademaker KJ, Koopman C, Groenendaal F (2004). Ultrasound abnormalities preceding cerebral palsy in high-risk preterm infants. J Pediatr.

[51] Himpens E, Oostra A, Franki I, Vansteelandt S, Vanhaesebrouck P, den Broeck CV (2010). Predictability of cerebral palsy in a high-risk NICU population. Early Hum Dev.

[52] Kuban KC, Allred EN, O’Shea TM, Paneth N, Pagano M, Dammann O (2009). Cranial ultrasound lesions in the NICU predict cerebral palsy at age 2 years in children born at extremely low gestational age. J Child Neurol.

[53] Leijser LM, De Bruine FT, Van Der GJ, Steggerda SJ, Walther FJ, Wezel-Meijler G (2010). Is sequential cranial ultrasound reliable for detection of white matter injury in very preterm infants?. Neuroradiology.

[54] Leijser LM, Liauw L, Veen S, de Boer IP, Walther FJ, Wezel-Meijler G (2008). Comparing brain white matter on sequential cranial ultrasound and MRI in very preterm infants. Neuroradiology.

[55] Levene MI (1981). Measurement of the growth of the lateral ventricles in preterm infants with real-time ultrasound. Arch Dis Child.

[56] Twomey E, Twomey A, Ryan S, Murphy J, Donoghue VB (2010). MR imaging of term infants with hypoxic-ischaemic encephalopathy as a predictor of neurodevelopmental outcome and late MRI appearances. Pediatr Radiol.

[57] Woodward LJ, Anderson PJ, Austin NC, Howard K, Inder TE (2006). Neonatal MRI to predict neurodevelopmental outcomes in preterm infants. N Engl J Med.

